# Review of Vibration Control Strategies of High-Rise Buildings

**DOI:** 10.3390/s22218581

**Published:** 2022-11-07

**Authors:** Mohamed Hechmi El Ouni, Mahdi Abdeddaim, Said Elias, Nabil Ben Kahla

**Affiliations:** 1 Department of Civil Engineering, College of Engineering, King Khalid University, P.O. Box 394, Abha 61411, Saudi Arabia; 2 LARGHYDE Laboratory, Civil Engineering and Hydraulics Department, Faculty of Science and Technologies, University of Biskra, Biskra 07000, Algeria; 3 Construction and Engineering Management, University of Twente, 7522 NB Enschede, The Netherlands

**Keywords:** vibration control, natural hazards, high-rise structures, passive control, active control, semi-active control

## Abstract

Since the early ages of human existence on Earth, humans have fought against natural hazards for survival. Over time, the most dangerous hazards humanity has faced are earthquakes and strong winds. Since then and till nowadays, the challenges are ongoing to construct higher buildings that can withstand the forces of nature. This paper is a detailed review of various vibration control strategies used to enhance the dynamical response of high-rise buildings. Hence, different control strategies studied and used in civil engineering are presented with illustrations of real applications if existing. The main aim of this review paper is to provide a reference-rich document for all the contributors to the vibration control of structures. This paper will clarify the applicability of specific control strategies for high-rise buildings. It is worth noting that not all the studied and investigated methods are applicable to high-rise buildings; a few of them remain limited by many parameters such as cost-effectiveness and engineering-wise installation and maintenance.

## 1. Introduction

**Figure 1:**
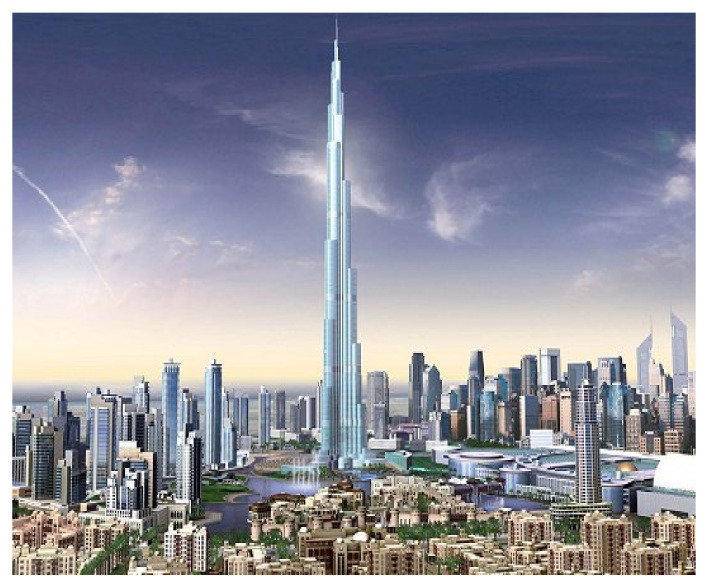
Borj Dubai (Dubai, 2008) [1].

In the past few decades, the design and construction of civil structures showed a deep evolution because of the technological progress in materials and devices. Buildings are getting taller, such as Borj Dubai (Figure 1), which is the tallest tower in the world at 828 m, and the new challenge today is the 1 Km Tower in Jeddah, KSA. High-rise buildings are becoming slenderer and more flexible, making them sensitive to the vibrations that natural risks cause (wind and earthquakes). As a result, controlling vibration is now a crucial concern in civil engineering.

The main purpose of vibration control is to prevent resonance, large amplitude oscillations, and unstable vibrations, as well as the quick suppression of transient vibrations. Numerous various forms of damping systems, each utilizing a different technical advancement, have been developed as a result of research in vibration suppression. The four categories of vibration control strategies are passive, active, semi-active, and hybrid.

Natural damping in flexible structures is frequently quite low, ranging from 0.1 percent to 5 percent; yet, even a modest increase in modal damping by the use of external dampers, devices, or damping materials may result in an acceptable reduction in response Høgsberg Høgsberg [2]. This implies that the introduction of the so-called passive dampers is perfectly adequate in conditions where the response is primarily controlled by resonance. Passive techniques do not need any energy source and can be classified into three categories, namely aerodynamic, structural, and mechanical vibration control. Many scholars have examined, both theoretically and experimentally, how well passive systems operate. Jangid and Datta Jangid and Datta [3] reviewed the seismic behavior of isolated buildings. Housner et al. [4] gave a thorough analysis of structural control’s past, present, and future using various control techniques. Buckle Buckle [5] evaluated the effectiveness of the passive control of seismically vulnerable constructions. Soong and Spencer Jr Soong and Spencer Jr [6] provided a cutting-edge analysis of how additional energy dissipation performs against natural risks. Kunde and JangidKunde and Jangid [7] examined how base-isolated bridges performed when subjected to dynamic forces. Spencer Jr and NagarajaiahSpencer Jr and Nagarajaiah [8] gave a detailed analysis of the structural control schemes. Later, Patil and Reddy Patil and Reddy [9] reviewed the effectiveness of base isolation systems applied in structures. Saaed et al. [10] gave a contemporary analysis of structural control techniques. 

## 2. Passive Control of High-Rise Buildings

**Figure 2:**
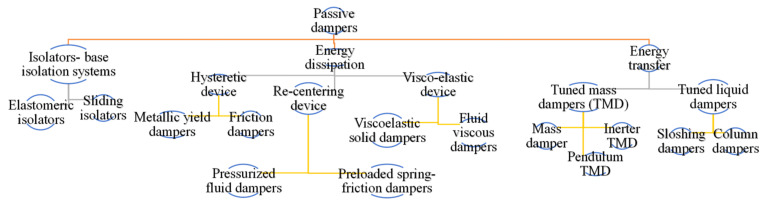
Division for passive damper.

Figure 2 shows different forms of passive dampers. Most of them have already been practically used in real-life high-rise buildings. 

### 2.1. Isolating Base or Energy Transfer?

This method consists in installing mechanical devices at certain locations along the structure or adding damping materials that control the structure vibrations without any energy source. 

Passive control has been widely practiced in civil engineering; the most used ones are:

#### 2.1.1. Base Isolation System

**Figure 3:**
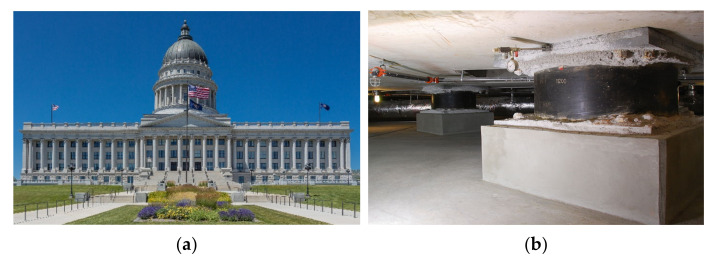
Utah State Capitol building (**a**) and the seismic dampening widgets (base isolators, (**b**)).

**Figure 4:**
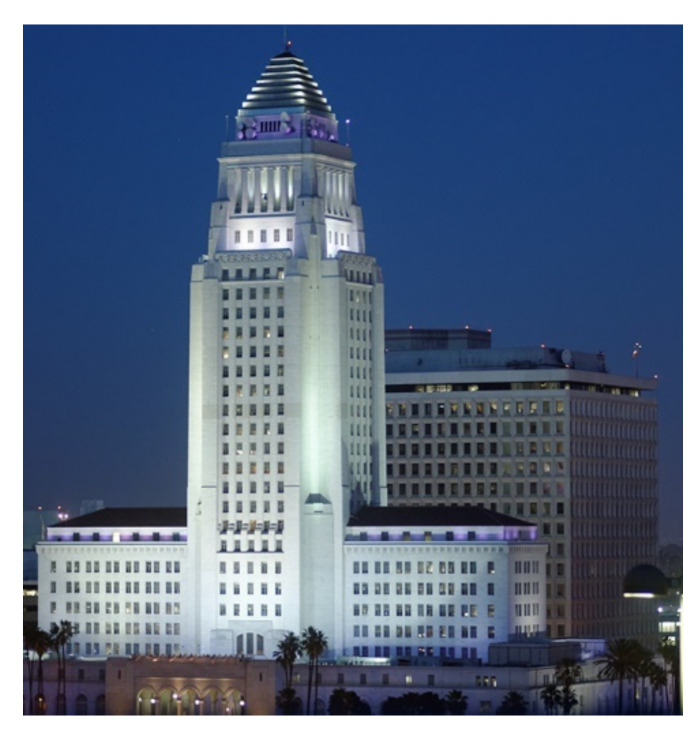
Los Angeles City Hall (base-isolated).

The installation of devices that decouple the structure or its main elements from potential hazardous earthquake-induced ground vibrations or support motions is the basic concept behind base isolation, also known as seismic isolation. This uncoupling is achieved by increasing the system’s flexibility and providing appropriate damping. This may be done using a laminated rubber (elastomeric) bearing base isolator with or without lead [11]. Base isolation can also be achieved using friction pendulum seismic isolation bearings [11,12,13,14,15,16,17,18] at each support point; this makes the structure sway with a gentle pendulum motion during earthquake ground shaking. This allows the ground to shake without damaging the structure. Friction isolation devices, yielding steel energy-absorbing devices [19,20,21] and visco-elastic [22,23] and fluid-viscous damping devices [24], are also used for base isolation. Mostly, the base isolation technique is used for low- to medium-rise buildings for seismic response mitigation [25,26,27,28,29,30,31,32,33,34]. However, recently, the application of isolation is proposed for high-rise buildings [35,36,37,38,39,40,41]. A detailed review of the base isolation system was recently provided by Beirami Shahabi et al. [42]. In addition, the base isolation techniques are practically used in real-life structures worldwide. Several buildings are already isolated around the world, and the greatest examples are the Utah State Capitol building and Los Angeles City Hall (see Figure 3 and Figure 4). 

#### 2.1.2. Tuned Mass Damper (TMD)

The dynamic vibration absorber (DVA) was first introduced in 1928 by Hartog and Ormondroyd Hartog and Ormondroyd [43] in mechanical engineering. Then, Den Hartog Den Hartog [44] provided the procedure for optimization of the DVA. Lenzen Lenzen [45] proposed the DVA in civil engineering under the name of TMD. It uses a secondary mass (usually concrete or steel) attached to a vibrating structure by a spring and a viscous damper. The characteristics of the dashpot, spring, and mass ratio are optimized with the aim of producing maximum damping. In essence, the TMD can be seen of as an energy sink where extra energy that has accumulated in the deck and pylons of the building or bridge is transmitted to a secondary mass. The building and the TMD mass itself are connected, and some sort of viscous damping device is used to absorb the energy.

**Figure 5:**
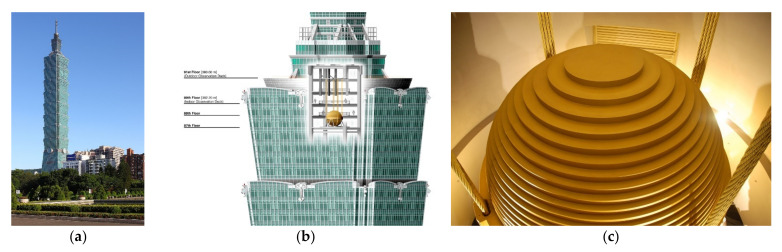
(**a**) Tower of Taipei 101 in Taiwan https://upload.wikimedia.org/wikipedia/commons/1/1a/Taipei_101_2009_amk-EditMylius.jpg (accessed on 15 October 2022); (**b**) TMD installed in the top of the tower https://upload.wikimedia.org/wikipedia/commons/1/15/Taipei_101_Tuned_Mass_Damper.png (accessed on 15 October 2022); (**c**) zoom on the TMD https://upload.wikimedia.org/wikipedia/commons/4/4a/Tuned_mass_damper_-_Taipei_101_-_Wikimania_2007_0224.jpg (accessed on 15 October 2022).

The pendulum TMD has been installed in many tall buildings, such as Taipei 101 in Taiwan (see Figure 5). Elias and Matsagar [46] presented a detailed literature survey for the research development of TMDs. Two recent progresses in the optimal use of TMDs are (i) distributing the TMDs along the height of the building [47,48,49,50] (this approach was particularly proven to be effective for a high-rise 76-story benchmark building submitted to wind loadings [51]) and (ii) use of inertance to connect the TMD to ground or lower floors [52,53,54,55,56,57,58,59,60,61,62].

#### 2.1.3. Tuned Mass Damper Inerter 

In the last decade, a new device was introduced to the domain of vibration control in civil engineering. The device is denoted as an “inerter” and has the capacity of producing a force relative to the acceleration between its terminals [53,63]. This force is equivalent to a fictive mass and can be used to enhance the performance of tuned mass dampers without increasing their vertical loads on the building frames; the resulting device is usually denoted a tuned mass damper inerter (TMDI) [64,65,66,67]. The association of the inerter to classical damping devices may result in other devices, such as the tuned inerter damper (TID), the multiple tuned mass damper, or the double mass tuned damper inerter (DMTDI) [68,69,70]. 

**Figure 6:**
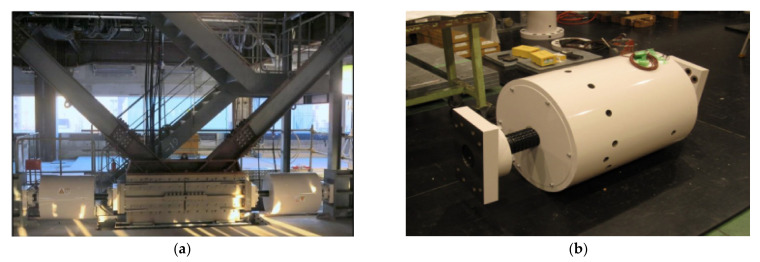
(**a**) Tuned viscous mass damper coupled to a chevron bracing equipping a building in Sendai, Japan; (**b**) tuned viscous mass damper device.

Despite the growing interest for the inerter applications in civil engineering, the majority of the research remains in the numerical simulation stage. However, a few real application to tall buildings can be found. Hence, a building in Sendai, Japan was equipped with a tuned viscous mass damper (TVMD) consisting of a viscous damper and a ball screw inerter in parallel connected to a spring in series [71]. The TVMD system is located in the upper floors of the building and aims to enhance the seismic safety of the structure. Both the TVMD and its location within the structure frame can be seen in Figure 6a,b. 

#### 2.1.4. Tuned Liquid Damper (TLD)

**Figure 7:**
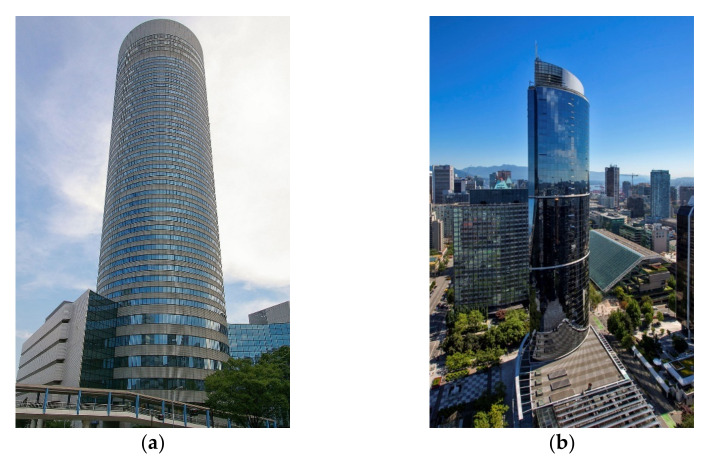
(**a**) Shin Yokohama Prince Hotel https://upload.wikimedia.org/wikipedia/commons/thumb/7/70/Shin_Yokohama_Prince_Hotel_20080808-002.jpg/375px-Shin_Yokohama_Prince_Hotel_20080808-002.jpg (accessed on 15 October 2022), Japan; (**b**) One Wall Centre in Canada (TLCD) https://upload.wikimedia.org/wikipedia/commons/thumb/7/73/One_Wall_Centre.jpg/375px-One_Wall_Centre.jpg (accessed on 15 October 2022).

TLD is a dynamic vibration absorber that uses the motion of shallow liquid (sloshing) in a partially filled container to dissipate the vibration energy. The geometry of the tank that holds the liquid and its viscosity is selected to have a frequency close to the fundamental frequency of the structure [72,73,74,75]. Utilizing flow-damping elements like screens or posts inside the container may cause the sloshing liquid’s energy dissipation to increase. Various container forms, such as rectangular or circular, can be used as TLD. Besides a circular type, a rectangular type has two different frequencies in two orthogonal directions [74,76]. For illustration, the Shin Yokohama Prince Hotel in Yokohama, Japan, is equipped with tuned liquid dampers, as shown in Figure 7a [77,78]. 

#### 2.1.5. Tuned Liquid Column Damper (TLCD)

This passive damping system uses water or other liquids in combination with the functions of the mass, spring, and viscous damping elements. The desired natural frequency of water motion is tuned by the geometry of the tank that holds the liquid [79,80,81]. A sluice gate device may be used to dissipate the energy in the moving water. The benefits of using a TLCD to reduce the motions of a building can be threefold [79]. A TLCD system is already implemented in the tower of One Wall Centre in Vancouver, BC, Canada as shown in Figure 7b. To reduce wind-induced vibrations, two tuned liquid column dampers (TLCDs) were developed. A 4-story high, 50,000-gallon (230-ton) water tank set to the appropriate frequencies constitutes each TLCD. Konar and Ghosh [75] presented a thorough analysis of the vibration control using several types of tuned liquid dampers.

### 2.2. Energy Dissipation 

This technique is one of the most practical and most used for structures with low to medium height. Here, the studies with practical usage in high-rise buildings are discussed. Some of the famous vibration mitigation methods in this area are discussed below. 

#### 2.2.1. Impact Dampers

**Figure 8:**
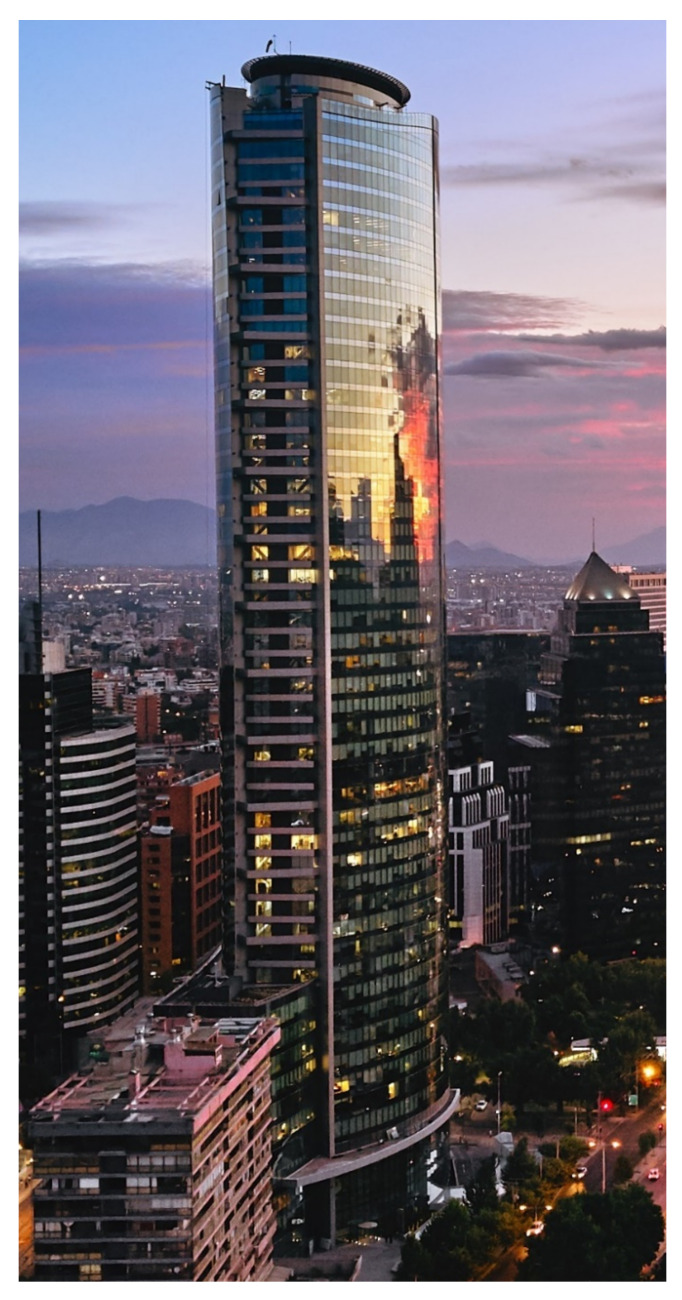
Titanium La Portada Building: https://upload.wikimedia.org/wikipedia/commons/thumb/7/74/Titanium_La_Portada_%2838888739395%29.jpg/360px-Titanium_La_Portada_%2838888739395%29.jpg (accessed on 15 October 2022).

Impact dampers [82,83] are an inertial system that consists of small rigid masses suspended from the top of a container mounted at its side to the structure. The container is designed with respect to an optimal spacing between the suspended mass and the container. The collisions between them dissipate the vibration energy from the structure. Lu et al. [84] presented a detailed literature survey on impact dampers. It has already been used in real-life structures; a good example is the Titanium La Portada [85] (see Figure 8).

#### 2.2.2. Passive Viscous Control Strategies

Passive control of vibration can also be achieved using an auxiliary structure. This strategy consists of connecting two parallel structures by passive devices (viscous dampers) [86,87].

**Figure 9:**
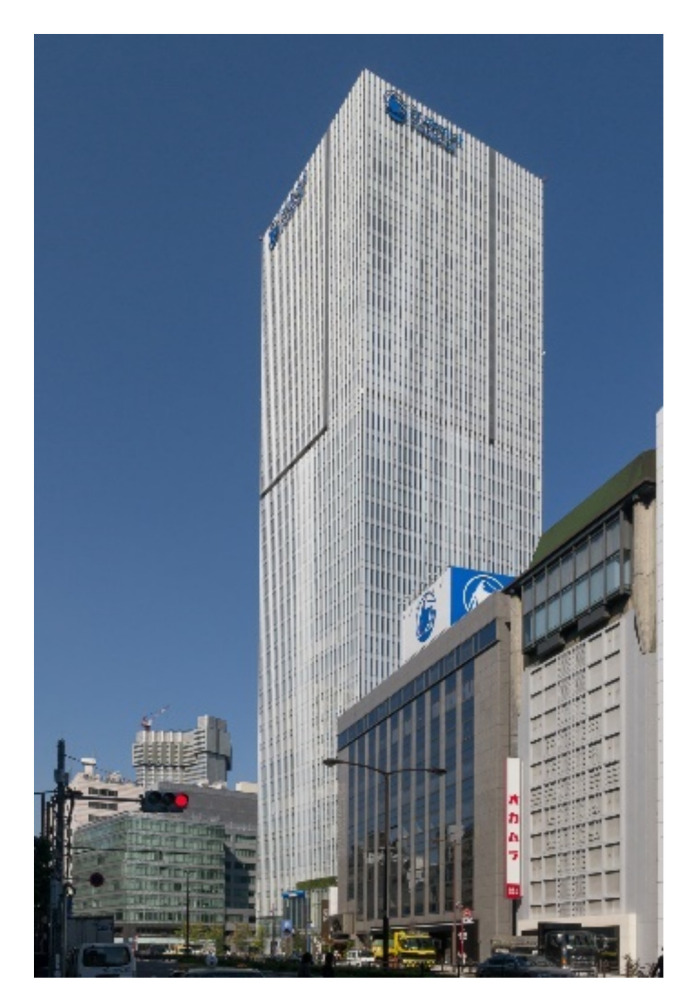
Prudential Tower in Tokyo https://upload.wikimedia.org/wikipedia/commons/3/37/Prudential-Tower-Tokyo-01.jpg (accessed on 15 October 2022).

Viscous brace systems (shear control) have also been used to control structure vibrations [88]. It has been installed in many buildings, such as Prudential Tower in Tokyo, as shown in Figure 9. A detailed literature survey of passive viscous dampers is provided by De Domenico et al. [89]. 

It is worth noticing that several studies investigated the performance of viscous dampers as coupling devices used to connect adjacent structures. Hence, Bhaskararao and Jangid [90] used viscous dampers to connect adjacent buildings. It was found that the dynamical response of both the connected structures was reduced. A large set of studies investigating the effect of viscous dampers as coupling devices can be found in the literature; numerically, this strategy has show its effectivness for high-rise buildings [91,92,93,94,95,96,97,98,99]. 

#### 2.2.3. Aerodynamic Control

**Figure 10:**
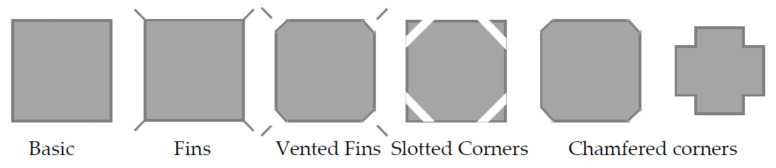
Examples of aerodynamic modifications to square building shapes.

**Figure 11:**
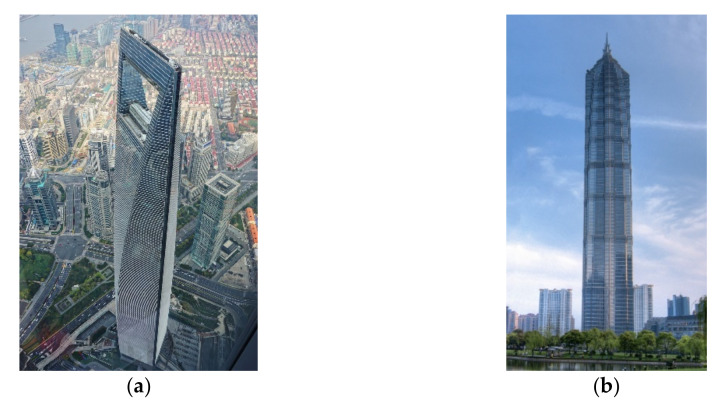
(**a**) Shanghai World Financial Center https://en.wikipedia.org/wiki/File:%E4%B8%8A%E6%B5%B7%E5%9B%BD%E9%99%85%E9%87%91%E8%9E%8D%E4%B8%AD%E5%BF%83.jpg (accessed on 15 October 2022). (**b**) Jin Mao towers https://en.wikipedia.org/wiki/File:Jin_Mao_Tower_2007.jpg (accessed on 15 October 2022).

The aerodynamic control of vibrations is performed by the modification of the cross-sectional configuration of the structure. For tall buildings, aerodynamic modifications (see Figure 10) include tapering and drop-off corners, slotted and chamfered corners, setbacks, fins, horizontal and vertical through-building openings, and sculptured building tops (see Figure 11). This passive technique has been used in several tall buildings, such as the Shanghai World Financial Center and Jin Mao Tower.

#### 2.2.4. Structural Control

**Figure 12:**
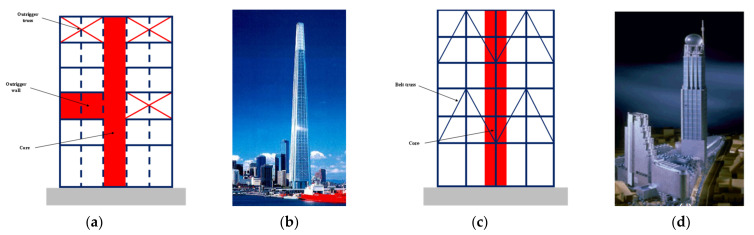
(**a**) Illustration of outrigger system; (**b**) Melbourne Tower; (**c**) illustration of “virtual outrigger” system using belt trusses; (**d**) Plaza Rakyat tower (Malaysia).

This strategy includes creating systems like space frames and mega-frame systems, as well as the addition of Vierendeel frames, belt trusses, super columns, bandages of the Vierendeel type, and outrigger trusses or walls. Concrete or composite steel/concrete construction with greater internal dampening might also be advantageous for a structural system. For example, Melbourne Tower, shown in Figure 12a,b, features 2-story deep outrigger trusses every 20 stories to aid in carrying lateral loads. This overcomes the restrictions facing core systems by transferring some of the loads to the exterior frame.

The Plaza Rakyat office tower in Kuala Lumpur uses belt trusses (Figure 12c,d). A concrete shear core and 2-story outer belt walls that are joined to the concrete perimeter frame at two levels make up the construction. By using standard outrigger systems, the lateral loads are carried without being constrained by mechanical space.

**Figure 13:**
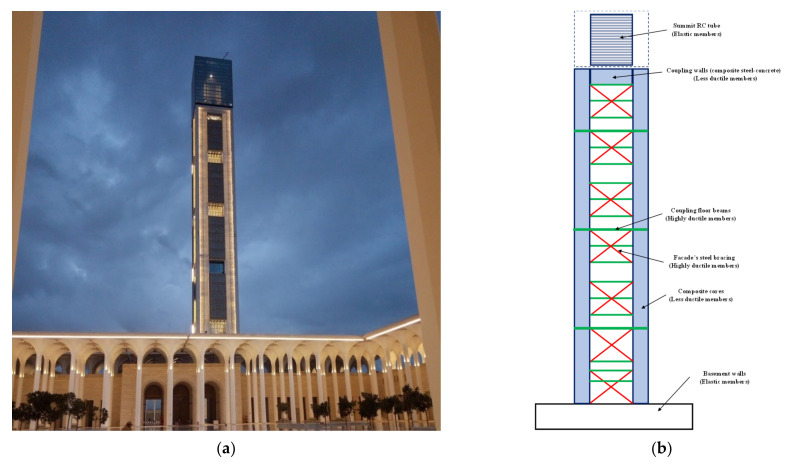
(**a**) General view of the Great Mosque of Algeria; (**b**) different structural members with respect to their design behavior.

Another example of structural control can be observed in the structural disposition of the minaret of the Great Mosque of Algeria, as can be seen in Figure 13a. The minaret is a slender structure with a height of 265 m above the ground and a square plan having a 26.8 m length on each side. The composite structure comprising four RC cores at the corners, slabs with an RC beam girder, and a stiffening system of steel profiles integrated in the RC cores provide the rising structure’s stiffening system. Due to the height seismicity of Algiers, the steel bracings were made as energy-dissipating components that yield before the foundation fails, in accordance with the aseismic strategy of capacity design. Three different types of earthquake-resisting systems can be differentiated in the load-bearing structure, namely, highly dissipative members, less dissipative members, and elastic members, as can be seen in Figure 13b [100,101,102]. 

Passive dampers are by definition dissipative and steady, making them dependable and resilient, but since they only affect the target frequency, they are unable to respond to changes in the environment, the structure, or the loading. Additionally, passive dampers do not work well when there are particularly strong earthquakes. As a result, active control, a new category of vibration control, has emerged.

## 3. Active Control

This strategy uses a set of actuators and sensors connected by a feedback or feedforward loop. 

**Figure 14:**
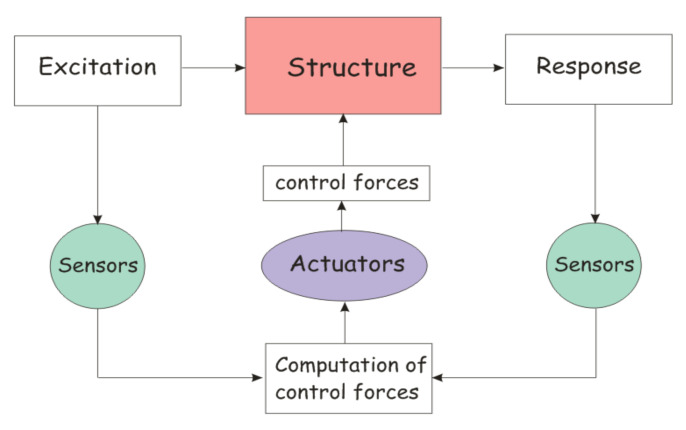
Block diagram of active control.

It has the basic configuration shown in Figure 14. In fact, civil structures’ vibrations can be controlled utilizing hydraulic or electromechanical actuator systems powered by a suitable control algorithm, such as closed-loop or feedback, where the control forces are determined by the structure’s feedback response, open-loop or feedforward, where the control forces are determined by measured external excitations, or closed-open loop or feedforward-feedback, where the control forces are determined by both the structure’s measured response and measured. When structure parameters are unknown and are based on tracking error between the measured response and the observed response, a system based on the variation of closed-open loop control with a controller that can adjust the parameters of the system, called adaptive control, can be used [103]. 

In 1968, Zuk Zuk [104] was the first to present the early notion of active control. He distinguished between active control, which is designed to reduce structural motion by generating control forces. In 1972, active control of civil structures was first introduced by Yao [105] as a means of protecting tall buildings against storms and became the subject of intensive research subsequently. Many active control strategies have been proposed. The most common ones are:

### 3.1. Active Mass Damper (AMD)

**Figure 15:**
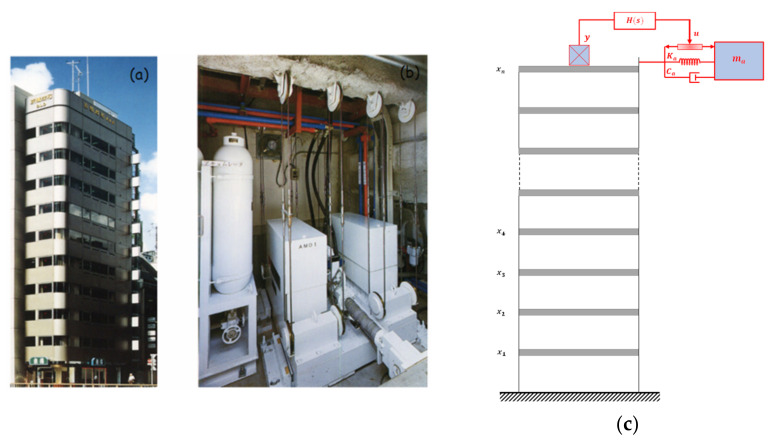
(**a**) Kyobashi Siewa Center (Japan) and (**b**) its AMD unit; (**c**) model of a building equipped with an AMD on the top floor.

This method generates a vibration control force in an actuator by using the reaction inertia force of an auxiliary mass (see Figure 15). In fact, a control computer analyzes measured response signals and applies a control force depending on the structural response’s feedback. To counteract the building motion, the actuator swings or pendulums the secondary mass. To reduce the vibrations of tall structures, Chang and Soong [106] proposed the active mass damper (AMD) in 1980 as an extension of a passive tuned mass damper (TMD). To effectively regulate a tall building exposed to stationary random wind forces, Abdel-Rohman [107] suggested a design procedure for an active TMD. Using an AMD, Samali et al. [108] evaluated the active vibration control of a 40-story building under significant wind excitations and contrasted the outcomes with a conventional TMD. Wu and Yang [109] suggested an AMD system to mitigate the vibrations in the Nanjing TV transmission tower in China based on linear-quadratic Gaussian (LQG), H∞, and continuous sliding mode control (SMC) strategies. The reaction mitigation capabilities of AMD systems installed in four actual steel-frame high-rise structures in Japan were presented by Yamamoto et al. [110]. Ikeda et al. [111] investigated the effectiveness of two AMD systems for controlling a building’s torsional and transverse vibrations. For buildings with seismic protection and AMD control systems, Wang and Li [112] proposed two controllers: fuzzy sliding mode control and variable structure control. Guclu and Yazici [113] examined the performance of PD and fuzzy logic controllers in controlling a 15-story frame supplied with AMDs on the first and fifteenth floors. The first full-scale application of active control to a building was accomplished by the Kajima Corporation in 1989 (Kobori et al. [114]). The control system installed on the Kyobashi Center building consists of two suspended AMDs (see Figure 15). The first AMD is used for transverse motion, while the second AMD is employed to reduce torsional motion. The two damper masses are driven by servo-hydraulic actuators [74]. Elias et al. [115] showed that the performance of the active controllers for vibration control of buildings subject to pulse-type ground motions is not significant. Plus, the assumption of elastic models is not realistic. Ümütlü et al. [116] showed the performance adaptive control design for an active TMD system. They found that the proposed system was robust under the dynamical forces. The absolute instantaneous optimal control performance index is as effective as LQR for an active control system in the response mitigation of structures [117,118,119]. 

### 3.2. Active Connected Building Control (CBC) Using Mutual Action between Structures

**Figure 16:**
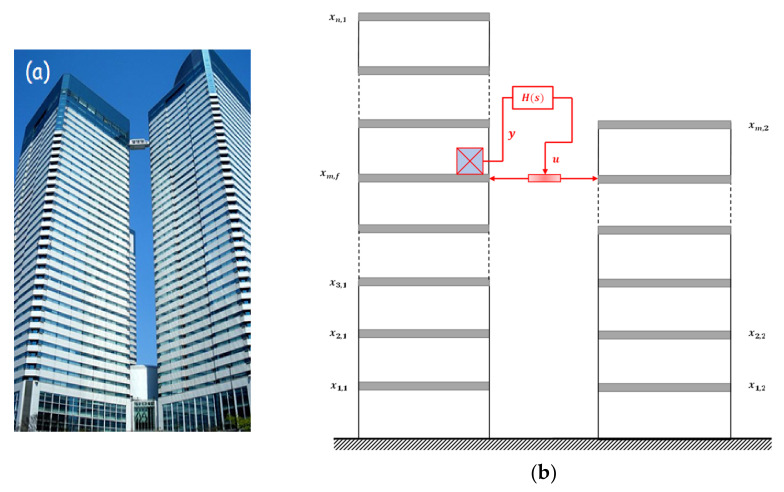
(**a**) Active CBC of Harumi Triton Square in Tokyo, Japan; (**b**) model of two adjacent buildings connected with an active strut.

The AMDs are used mainly to reduce the vibration of high-rise buildings caused by moderate earthquakes or strong wind; however, this strategy may not be effective with large-scale earthquakes or the very low frequency of ultra-tall towers. To solve these problems, active CBC can be used. As shown in Figure 16, this method generates a control force using an actuator positioned on a support structure that is parallel to the flexible structure. This approach has the advantage of obtaining adequate control force at low frequencies. It also provides more living comfortability and convenient living with interchange using a connecting bridge between the buildings [120]. The active CBC of tall buildings subjected to seismic excitation has been studied by Seto and coworkers [121,122], Yamada et al. [123], and Christenson et al. [124]. In those investigations, the linear-quadratic control approach was used to ascertain the control forces of coupled structures. The seismic response of connected buildings has also been reduced using the nonlinear optimum control method [125,126,127,128,129]. The stochastic optimal coupling control of nearby building structures was investigated by Ying et al. [130] on the basis of the stochastic dynamical programming principle and stochastic averaging method. The active CBC system is already installed in the Harumi Triton Square in Tokyo (Japan).

### 3.3. Active Bracing System (ABS)

**Figure 17:**
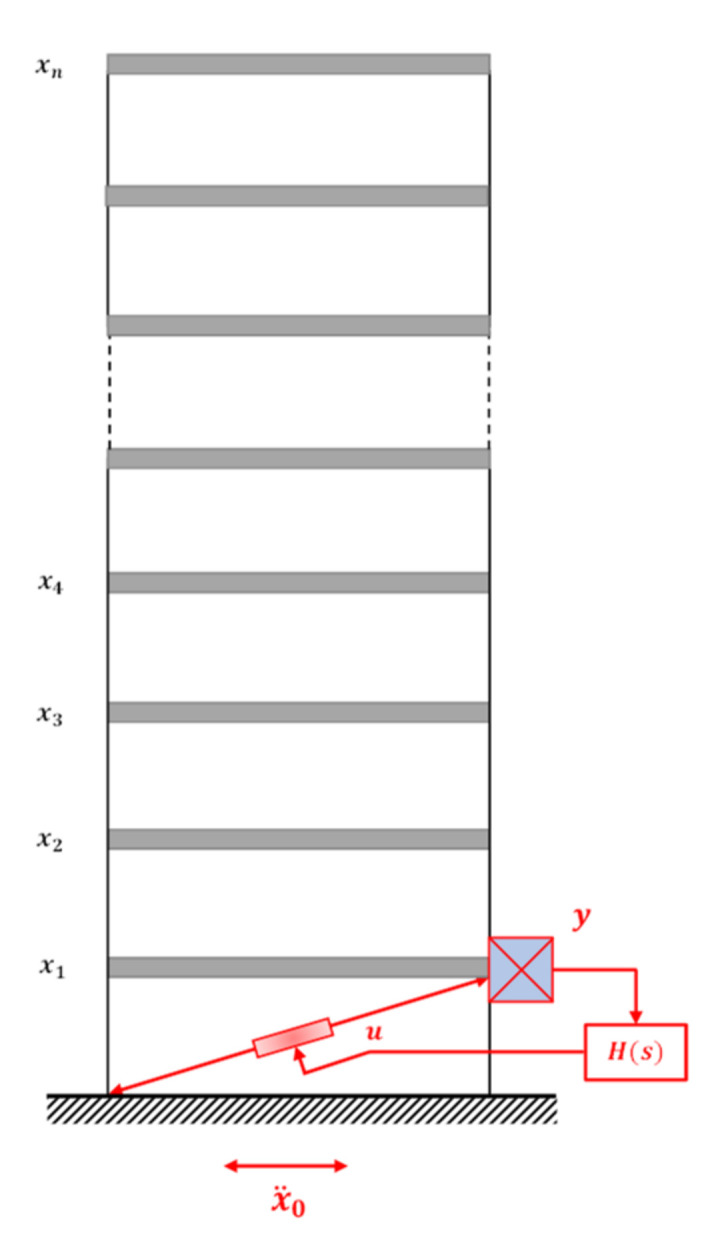
n-story shear frame equipped with an ABS between the ground and the first floor.

Active bracing systems, which provide active elements between two succeeding levels or between the ground and the first floor, as shown in Figure 17, can also reduce the vibration of buildings. According to the LQR theory, Chung et al. [131,132] used tendons attached to a servo-hydraulic actuator to operate a single-degree-of-freedom (SDOF) and 3DOF structures. The effectiveness of a few control algorithms was investigated by Loh et al. [133] using a full-scale, 3-story steel building with an active bracing system placed on the first floor. Three different control algorithms—static-output-feedback LQR control, modal control with direct output feedback, and static-output-feedback with variable gain—are used in the experimental verification. A discrete-time modal control scheme was put forth by Lu [134] and is very effective and shows potential for reducing the seismic response of building structures fitted with ABS.

### 3.4. Active Tendon Control

**Figure 18:**
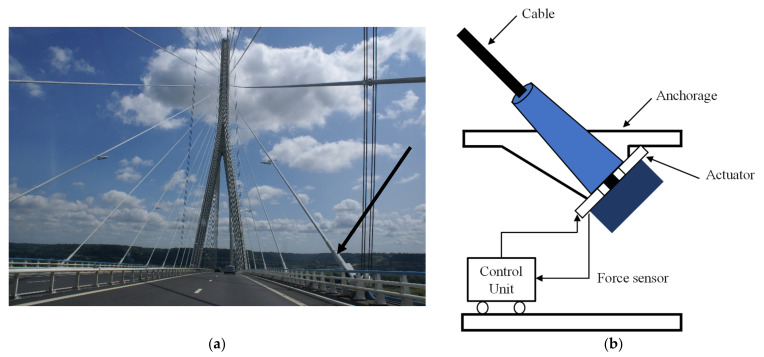
(**a**) Normandy Bridge equipped with active tendon cable https://upload.wikimedia.org/wikipedia/commons/c/cc/Pontdenormandie.JPG (access on 15 October 2022); (**b**) active tendon mechanism.

The system for buildings is made up of tendons that are attached to the proper location in the structures and electrohydraulic servomechanisms. To reduce the structure’s vibration, the hydraulic rams’ movements generate the control forces in tendons (Figure 18). The sensors and the control algorithm regulate the motion of the hydraulic rams. Active tendons are usually used in cable-stayed bridges and can be found in multiple bridges around the world. The most illustrative example can be observed in the Normandy Bridge, France; the span of the bridge is 850 m and it is equipped with both stay cables and control cables [135]. The main difficulty with such systems in the nonlinear behavior of cables, especially under combined loads of wind and passing vehicles. 

### 3.5. Active Control Algorithms

All active strategies described above use control algorithms [11]. Control algorithms select sensor and actuator placements; then, based on the measurements from sensors, the control gains are optimized, and the required control forces or displacements are computed. The common ones are:

#### 3.5.1. Linear Optimal Control

In linear control, all mathematical equations and operations are linear.

Consider the system:

$$\dot{x}=Ax+Bu;\;x(0)=x_{0}$$

where the system matrix *A* is not necessarily stable, but it is assumed that the pair (*A*, *B*) is controllable. The basic optimal algorithms are presented below:

• Linear quadratic regulator (LQR)

The LQR algorithm consists of the minimization of a quadratic cost functional *J* of the following form:

$$J=\int_{0}^{\infty }[x^{T}Q\;x+u^{T}R\;u]dt$$

where *Q* and *R* are referred to as weighting matrices and *u* = −*G z* is the control force (*G* is the gain matrix). 

The unknown external excitation is not considered throughout the reduction process. This control algorithm is not really optimal as a result. In actuality, a large number of control algorithms are not really optimal in this regard. Numerous studies on linear optimum control have been conducted [106,132,136,137,138,139].

• *H*2 control

*H*2 control is an idealized control based on the 2-norm that the mean can identify more easily in one-dimensional space [140]. Recall:

$${\left\|x\right\|}_{2}={\left(\sum_{i=1}^{N}{\left|x_{i}\right|}^{2}\right)}^{1/2}$$

Control is beneficial for lowering the overall root mean square response for structural control applications. We have the following for the state-space system *G*(*s*):

$${\left\|G(s)\right\|}_{2}≃\sqrt{\frac{1}{2\pi }\int_{-\infty }^{\infty }tr(G^{H}(j\omega )G(j\omega ))d\omega }$$

Therefore, the system’s *H*2 is comparable to the minimizing of all singular values or harmonic frequencies in terms of physics. The *H*2 control can be helpful in guaranteeing overall structural integrity to protect against seismic excitation in this way [141,142]. 

• *H∞* Control

Another form of optimum control based on the infinity-norm is *H∞* control. Recall:

$${\left\|x\right\|}_{\infty }=\max(\left|x_{i}\right|)$$

As a result, the *H∞* control aims to reduce the highest response possible in a structure [143]. Relating the *G*(*s*) state space system to the *H∞* norm:

$${\left\|G(s)\right\|}_{\infty }\underset{\omega }{≜\max\bar{\sigma }}(G(j\omega ))$$

In terms of physics, the *H* norm merely determines the system’s maximum value. Since unstructured model uncertainties are conveniently represented by the *H∞* norm, structural control applications can benefit greatly from its use. Furthermore, the *H∞* norm’s virtue of being an induced norm makes it simpler to implement in big systems than the *H*2 norm.

#### 3.5.2. Instantaneous Optimal Control

In the instantaneous optimum control, the control algorithm based on the time-dependent performance function *J* is improved using the information of external excitation up to the current time (t). The adequate control force is calculated by minimizing *J*(*t*) at any instant of time, *t*. Writing the state vector z(t) in terms of the state vector and at the prior time step, which is assumed to be known, is how the problem is formulated. Over the time span Δ*t* the cost function is reduced. Abdel-Rohman and Leipholz Abdel-Rohman and Leipholz [144] and Yang et al. [145] have made noteworthy contributions to instantaneous control. 

#### 3.5.3. Sliding Mode Control (SMC)

Utkin [146] was the first to introduce the sliding mode control (SMC) scheme. This algorithm consists in generating a sliding surface with a linear combination of state variables, such that the motion of the structure is stable on this surface. The sliding surface is obtained by minimizing a performance function of LQR type and thus by solving the Riccati equation. Then, based on the Liapunov stability criterion, controllers are designed to drive the response trajectory onto the sliding surface. An improvement over the SMC is achieved by designing a controller based on a linear feedback system and nonlinear feedback of the state vector. To make the control strategy more robust, the nonlinear feedback system takes into account the uncertainties arising from the excitation (see Yang et al. [147] and Sarbjeet and Datta Sarbjeet and Datta [148]).

#### 3.5.4. Nonlinear Control 

The nonlinear control consists of minimizing a higher-order performance function such that the control force becomes a nonlinear function of the state variable. Wu et al. [149] developed a nonlinear control strategy based on the LQR and the solution of the Riccati equation. The control force was expressed in a convenient form by using a weighted nonlinearity feedback parameter. Other works on nonlinear active control include those of another type of nonlinear control scheme presented by Shefer and Breakwell Shefer and Breakwell [150] and Suhardjo et al. [151] for the response reduction in nonlinear structures. The control algorithm takes into account stiffness, and damping nonlinearities and the nonlinear equation of motion can be solved in the time domain, with a control force derived as a nonlinear function of the state variable. The minimization of the nonlinear performance function is again achieved through the solution of the Riccati equation.

#### 3.5.5. Active Control Using Neural Network and Fuzzy Logic

Fuzzy logic theory and neural networks’ primary goal is to avoid the need for creating a control algorithm analytically. These control systems are superior in terms of practical applications and are more adaptable, even though they do not rigorously and ideally manage the structural response. Neural networks are commonly used in active control of structures to offer control forces that lessen the response of the structure to future earthquakes that are unknown [152,153,154,155].

The fuzzy control method is reliable and capable of handling the structure’s nonlinear behavior. Additionally, the calculations required to drive the controller are fairly straightforward and may be simply included into a fuzzy chip. The Simulink and fuzzy toolboxes in the MATLAB environment are typically used to solve the control equation of motion. 

By taking into account feedback as (i) only velocity, (ii) velocity and displacement, and (iii) velocity, acceleration, and displacement, different forms of fuzzy rule bases can be utilized to map control forces according to the levels of the structural responses. Although fuzzy control does not offer optimal control, it is more flexible than traditional control theories. Battaini et al. [156], Kurata et al. [157], and Tani et al. [158] have all described various applications of fuzzy control theory to various sorts of structures.

#### Wavelet-Based Control Algorithm

The concept of wavelets was first introduced to the vibration control of structures by Adeli and Kim [159]. By effectively combining a feedback control algorithm, such as the LQR or LQG algorithm, the filtered-x (LMS) algorithm, and wavelets, they developed a new wavelet-hybrid feedback least mean square (LMS) algorithm for resilient management of civil structures [160]. The new wavelet-based control technique has a number of benefits, including formulating the external excitation term, suppressing vibrations across a spectrum of input excitation frequencies, and being less vulnerable to modeling errors. Ref. [161] Utilizing a wavelet-based control technique, the active vibration control of cable-stayed bridges subject to seismic excitations was examined.

#### Other Active Control Laws

Other active control laws have been introduced for structural damping in space and mechanical applications and may be good candidatures for civil engineering applications, such us:

- First and second-order positive position feedback (PPF), which uses a force actuator and displacement sensor [162,163,164].
- Integral force feedback (IFF) [165], which uses a force sensor and a displacement actuator.
- Direct velocity feedback (DVF) [166], which uses a force actuator and a velocity or acceleration sensor.
- Lead compensator [164], which uses a force actuator and a displacement sensor.
- Proportional integral derivative (PID) controller [166], which uses control loop feedback to control process variables such as displacement, velocity, and acceleration.

Generally, active control has high performance on the target mode and also acts indirectly on other modes, but the stability is guaranteed only if the sensors are collocated with the actuators (physically located in the same place), and, in some cases, it is subject to spillover instability, which means that the control system always tends to destabilize the flexible modes just outside the control bandwidth (residual modes). Another disadvantage of active control is the requirement of energy, which implies the high cost of this strategy. All the active control algorithms, laws, and strategies presented in this section may be used in a semi-active control version.

### 3.6. Semi-Active Control

To reduce the high cost of active control, a new control method called semi-active, which combines the best features of both passive and active strategies, was introduced by Karnop et al. in 1973 in mechanical engineering [167] and by Hrovat et al. [168] in 1983 in civil engineering. Semi-active control devices give the adaptability of active control devices with a minimum of energy and may run on battery power, which is crucial during seismic events when the primary power source to the structure may fail. This has attracted attention in recent years. A semi-active control device has qualities that can be modified to best reduce the system responses but cannot inject mechanical energy into the controlled structural system (containing the structure and the control device) [169]. According to preliminary research, semi-active systems outperform passive devices when implemented properly, opening the door to the possibility of successful response reduction under a variety of dynamic loading scenarios [169,170,171,172]. There are many semi-active systems available, including electro-mechanical devices, smart tuned mass dampers, tuned liquid dampers, controllable friction devices, controllable fluid dampers, and controllable stiffness devices.

#### 3.6.1. Variable-Orifice Dampers

**Figure 19:**
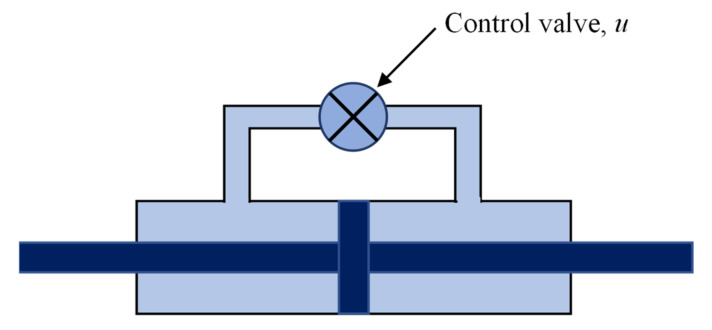
Variable-Orifice Dampers.

This device modifies a traditional hydraulic fluid damper’s resistance to flow using a programmable, electromechanical, variable-orifice valve [8] (Figure 19). This concept was first proposed by [173] to control the motion of bridges experiencing seismic motion, and later it was studied analytically and experimentally by a number of researchers, including Kawashima and Unjoh Kawashima and Unjoh [174], Patten et al. [175], and Symans and Constantinou Symans and Constantinou [176]. To reduce traffic-induced vibrations, Sack and Patten Sack and Patten [177] created a hydraulic actuator with a variable orifice, which Neff Patten et al. [178] deployed in a full-scale bridge on Interstate Highway I-35 in Oklahoma. Symans and Kelly Symans and Kelly [179] have conducted analytical and experimental research on the use of variable fluid dampers for reducing structures’ seismic responses. An on-off controllable orifice hydraulic damper employed as a resettable stiffness device has been explored by Jabbari and Bobrow Jabbari and Bobrow [180] and Yang et al. [181]. Additionally, Mori Tower makes use of variable-orifice dampers (Tokyo).

#### 3.6.2. Variable-Stiffness System 

**Figure 20:**
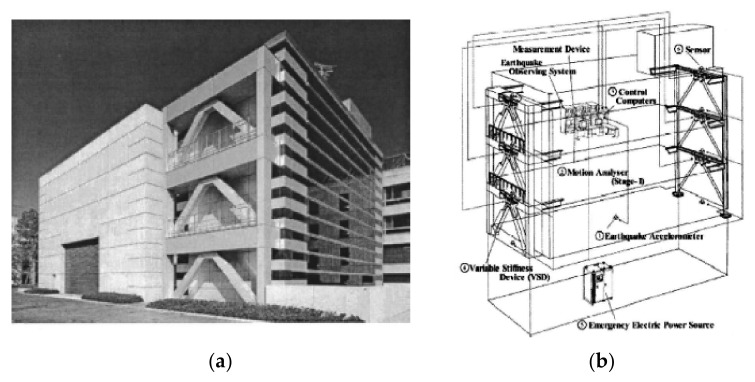
(**a**) Kajima Technical Research Institute with AVS system; (**b**) control scheme used in the Kajima Technical Research Institute [8].

The variable-stiffness system is composed of many variable-orifice dampers used to produce an on-off mode: (i) when the valve is closed, a very high stiffness can be achieved because of hydraulic fluid compressibility and (ii) when the valve is open, the stiffness becomes very small. The main drawback of these devices is that they cannot vary stiffness continuously between different stiffness states [8]. Kobori et al. [182] implemented a full-scale variable-stiffness system (AVS) to investigate semi-active control of the Kajima Research Institute Building (Figure 20). Nagarajaiah Nagarajaiah [183] has developed a semi-active continuously and independently variable-stiffness device (SAIVS). Nagarajaiah and Mate Nagarajaiah and Mate [184] have shown the effectiveness of the SAIVS device in a scaled structural model by varying the stiffness smoothly and producing a non-resonant system.

#### 3.6.3. Controllable-Fluid Device

**Figure 21:**
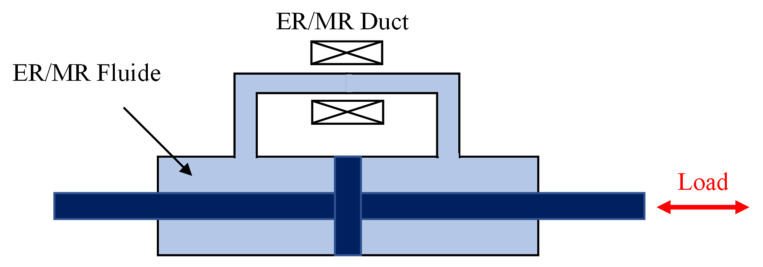
Controllable-fluid damper.

**Figure 22:**
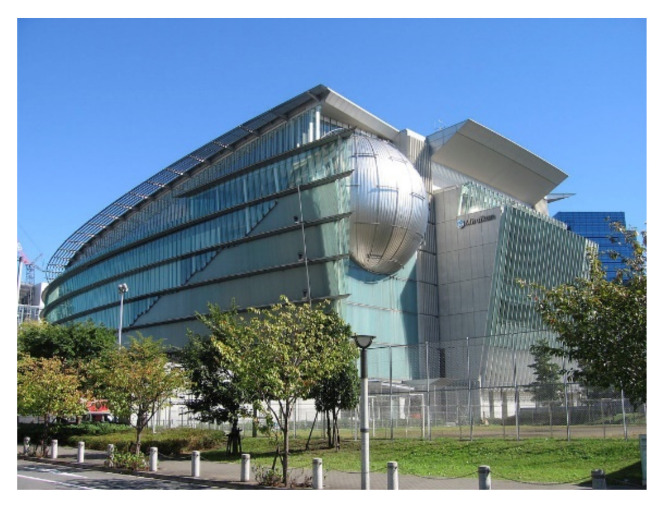
The National Museum of Emerging Science and Innovation (Tokyo) https://upload.wikimedia.org/wikipedia/commons/thumb/f/ff/Miraikan.jpg/1024px-Miraikan.jpg (access on 15 October 2022).

Electro-rheological (ER) or magneto-rheological (MR) fluids are used in controllable-fluid devices [8]. They are made of a fluid-filled hydraulic cylinder with microscopic dielectric particles (Figure 21). When there is current present, these particles polarize and increase flow resistance, converting viscous fluid into a yielding solid in milliseconds. Electromagnets situated inside the piston head of the MR dampers, which are magnetic counterparts of ER dampers, produce the magnetic field [185,186,187,188,189,190]. However, only MR fluids have been demonstrated to be tractable for applications in civil engineering [170]. At the Tokyo National Museum of Emerging Science and Innovation, about 30 MR fluid dampers were placed (Figure 22).

#### 3.6.4. Variable Friction Devices

Variable friction devices are based on forces generated by surface friction to dissipate vibratory energy in a structural system. The devices proposed by Akbay and Aktan Akbay and Aktan [191] and Kannan et al. [192] consist of a friction shaft that is rigidly connected to the structural bracing. The force at the frictional interface was adjusted by allowing slippage in controlled amounts. Feng et al. [193] have employed a semi-active friction controllable fluid bearing in parallel with a seismic isolation system. Yang and Agrawal Yang and Agrawal [194] have studied variable friction systems for seismic response reduction in nonlinear buildings. Garrett et al. [195] have studied piezoelectric friction dampers experimentally. 

#### 3.6.5. Electro-Mechanical Devices

**Figure 23:**
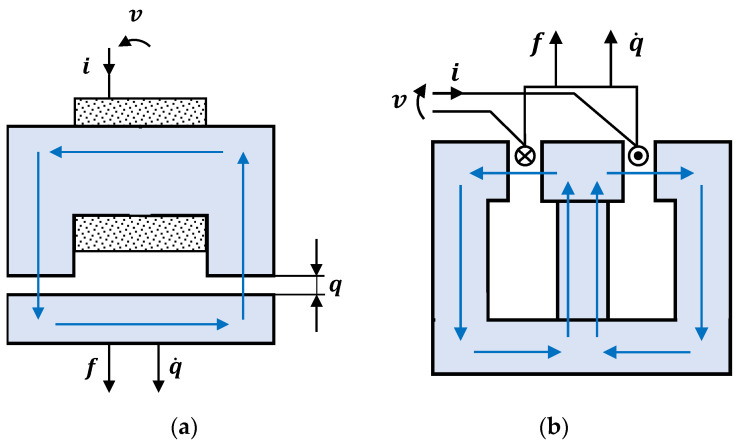
(**a**) Maxwell magnetic actuator and (**b**) Lorentz magnetic actuator.

Electro-mechanical devices are based on forces generated by the variation in the magnetic circuit’s reluctance that causes the magnetic flux linkage to vary over time; this is mainly observed in the Maxwell magnetic actuator (Figure 23a), while in the Lorentz magnetic actuator (Figure 23b) the force is the result of the interaction between eddy currents produced in a conductor traveling in a constant magnetic field. These two types of electro-mechanical devices are polyvalent and can be used as passive, active, or semi-active controllers [196]. Despite their excellent potential, electro-mechanical devices were not used to control the vibration in high-rise structures. However, they could be used with the development of technologies related to their exploitation and maintenance. 

#### 3.6.6. Semi-Active TMD and Semi-Active TLD

A TMD with changing stiffness is referred to as a semi-active tuned mass damper (STMD). Real-time control gives it the particular advantage of continuously returning to its frequency, making it resistant to changes in building stiffness and dampening [197]. By utilizing the SAIVS device, Nagarajaiah and Varadarajan Nagarajaiah and Varadarajan [198] created this device, and they have experimentally and analytically demonstrated its efficacy on a small-scale 3-story structural model.

In the TLDs, the sloshing frequencies of the fluid are changed by modifying the length of the hydraulic tanks and adjusting the rotation of the rotatable baffles in the tank. 

All the semi-active devices presented above had been also used with base isolation [199,200,201], CBC control [202,203], and ABS control [204]. 

#### 3.6.7. Semi-Active Impact Dampers

An impact damper is composed of a loose mass within the main mass connected to the host structure through a dashpot and a spring. This system has been proven to be very effective in reducing undesirable vibrations in mechanical systems [205]. Combined with a magnetorheological device, the impact damper results in a so-called semi-active impact damper or smart impact damper [206]. To the authors’ best knowledge, these control schemes were not exploited in controlling the response of high-rise buildings. 

### 3.7. Hybrid Control

Another type of control strategy is the hybrid device, which was also designed to overcome the shortcomings of a passive system that performs inadequately in connection with very large earthquakes. In the case of a TMD, the building may be equipped with a passive auxiliary mass damper system and a small tertiary mass connected to the secondary mass with a spring, damper, and an actuator (Duox). The secondary system is set in motion by the active tertiary mass, and it is driven in the direction opposite to the TMD, making it more effective [207,208].

**Figure 24:**
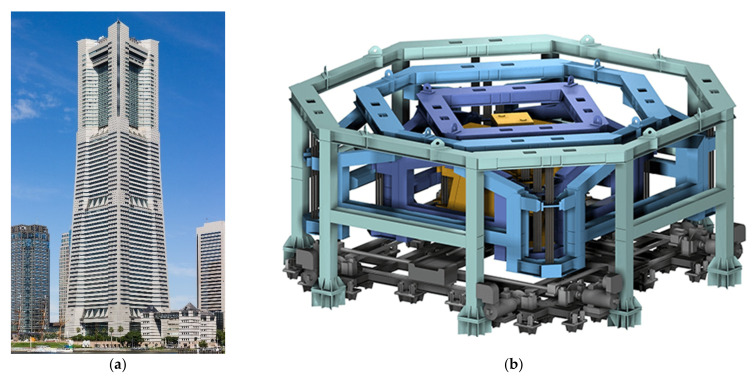
(**a**) Landmark Tower in Yokohama equipped with tow HMDs https://upload.wikimedia.org/wikipedia/commons/0/03/Yokohama_Landmark_Tower_201507.JPG (access on 15 October 2022); (**b**) HMD device https://www.mhi.com/products/infrastructure/images/steelstructures_vibrationcontrol_case07.png (access on 15 October 2022).

Hybrid mass dampers (HMDs) behave as either a TMD, using the concept of moving mass supported mechanisms of the same natural period as the building, or an AMD, according to the wind conditions and building and damper mass vibration characteristics (Tamura et al. [209]). The active portion of the system is only used in the case of large excitations. Otherwise, it behaves passively. The main advantage of these systems appears in the cases of power failure or extreme excitations that exceed the actuator capabilities; the HMD device will automatically switch into passive mode until the system can safely resume normal operations. HMD was installed in many tall buildings in the world, such as Landmark Tower in Yokohama (Figure 24a). The installed HMD device is represented in Figure 24b. 

Kim and Adeli Kim and Adeli [210] introduced a hybrid control system consisting of a passive supplementary damping system and a semi-active tuned liquid column damper (TLCD) system. They evaluate the effectiveness and robustness of the hybrid damper-TLCD system in reducing vibrations under various seismic 8-story frames using a new wavelet-based control algorithm [134]. 

## 4. Illustration of Literature Results 

To illustrate the efficiency of vibration control devices deployed in high-rise buildings, a review of various results obtained and presented in the literature is resumed in the two following tables [211,212,213,214,215,216,217,218,219,220]. Table 1 presents the results obtained when submitting high-rise benchmark buildings to earthquake excitation while equipping them with different control devices (best performances), and Table 2 shows the results of the same high-rise buildings submitted to wind excitations. The tables also show the device type, the quantity of interest, and the percentage reduction obtained when compared to the uncontrolled case. 

It can be seen from Table 1 and Table 2, the performances of control devices are extensively studied for high-rise buildings subjected to both earthquakes and wind loadings. It can be clearly stated that the performance of these devices can effectively reduce induced vibrations, henceforth reducing both structural damage and users’ discomfort. 

## 5. Conclusions

This survey paper presents an extensive view of the practical applications of damping devices and systems applied to high-rise structures subjected to dynamical loadings. It is worth noting that most vibration control devices were initially developed for mechanical problems and then adopted for civil engineering problems. Despite the large variety of devices and control strategies, a few found their way to real engineering applications. They can be identified in high-rise buildings such as tuned mass dampers, tuned liquid dampers, and bracing systems. The systems are the most used because of their cost effectiveness and relative ease of maintenance.

With the fast development of technologies such as 3D printing and artificial intelligence, it may be possible that other control devices and strategies such as semi-active dampers and active tendons will be introduced to mitigate the vibrations in high-rise buildings. 

With this in the background, it is worth saying that without the introduction of vibration control devices, many building designs would have failed under the numerous seismic events and strong winds recorded in different regions of the world. These vibration control devices are more than necessary nowadays with the growing ambitions of architects and engineers.

## Figures and Tables

**Table 1:** Performance of various control strategies for reducing earthquake-induced vibrations in high-rise buildings.

Researcher(s)	Used Device	N° of Floors	Reduction in Dynamical Parameters of Interest		
			Peak Displacement	Peak Acceleration	Peak Base-Shear
Elias and Matsagar [211]	TMD	20	20%	10%	10%
Elias et al. [220]	Multiple Distributed TMD	20	30%	40%	35%
Samiee [212]	TLD	20	17.42%	7.2%	/
Halperin et al. [213]	Viscous damper	20	30–42%	70–82%	/
Bitaraf and Hurlebaus [214]	MR damper	20	46%	55%	/
Raut and Jangid [215]	Friction damper	20	46.7%	23.5%	5.33%

**Table 2:** Performance of various control strategies for reducing wind-induced vibrations in high-rise buildings.

Researcher(s)	Used Device	N° of Floors	Reduction in Dynamical Parameters of Interest		
			Peak Displacement	Peak Acceleration	Peak Base-Shear
Banerjee et al. [216]	TMD	25	12.7%	21.8%	/
Suthar and Jangid [217]	TLCD	76	35.5%	38.8%	/
Koutsoloukas et al. [218]	HMD	15	23%	37%	/
Elias et al. [50]	Multiple Distributed TMD	76	56%	52%	
Li et al. [219]	TTMDI	25	/	42.81%	/
